# Editorial: Molecular mechanisms of treatment for immune dysregulation by targeting toll-like receptors

**DOI:** 10.3389/fmed.2024.1493523

**Published:** 2024-10-04

**Authors:** Juandy Jo, Johan Garssen

**Affiliations:** ^1^Department of Biology, Faculty of Health Sciences, Universitas Pelita Harapan, Tangerang, Indonesia; ^2^Mochtar Riady Institute for Nanotechnology, Tangerang, Indonesia; ^3^Division of Pharmacology, Utrecht Institute for Pharmaceutical Sciences, Faculty of Science, Utrecht University, Utrecht, Netherlands; ^4^Danone Nutricia Research, Utrecht, Netherlands

**Keywords:** toll-like receptor agonists, immunomodulation, therapeutic agents, adjuvants, molecular mechanism

The mammalian immune system is equipped with pattern-recognition receptors, including Toll-like receptors (TLRs), to recognize conserved structural motifs, including both pathogen- and danger-associated molecular patterns (PAMP and DAMP), resulting in protective immune responses against pathogens or damaged cells. The TLRs are type 1 transmembrane proteins, comprising an extracellular domain of leucine-rich repeats and cysteine-rich motifs and an intracellular domain of Toll/IL-1 receptor homology (TIR), in which the TIR domain is essential for signaling. To date there have been 10 human TLRs characterized, which can be categorized into two subclasses: cell-surface TLRs (TLR1, TLR2, TLR4, TLR5, TLR6, and TLR10) and endosomal TLRs (TLR3, TLR7, TLR8, and TLR9). Cell-surface TLRs primarily detect microbial components like lipids, lipoproteins and proteins. For example, TLR4 recognizes bacterial lipopolysaccharide (LPS), while TLR2, along with TLR1 or TLR6, recognizes various PAMPs like lipoproteins and peptidoglycans. In addition, TLR5 senses bacterial flagellin, while human TLR10 works with TLR2 to identify ligands from *Listeria* species and influenza A virus. In contrast, endosomal TLRs detect nucleic acids from bacteria and viruses, in addition to self-nucleic acids from damaged cells. For example, TLR3 recognizes viral double-stranded RNA (dsRNA), small interfering RNAs, and self-RNAs from damaged cells, while TLR9 detects unmethylated DNA with CpG-motifs. In addition, while TLR7 detects viral single-stranded RNA (ssRNA), human TLR8 responds to both viral and bacterial RNA. TLRs are predominantly expressed by cells of the innate immune system, including macrophages and dendritic cells. Non-immune cells, including endothelial and epithelial cells, also express various TLRs but to a lesser degree. Activation of TLRs would stimulate the innate immune system to release pro-inflammatory cytokines (e.g., tumor necrosis factor alpha (TNF-alpha), IL-12 and IL-18) and type I interferons, which would subsequently activate the adaptive arm of the immune system. Modulation of TLR signaling may be beneficial in both preventing and treating immune-related disorders, and this TLR modulation may be used as adjuvants or therapeutic agents.

Published articles within this Research Topic can be classified into two groups. The first group includes articles that study TLR activation and signaling to generate immune responses. Individuals with weak immune responses are prone to developing life-threatening infections. Trained immunity is a strategy that boosts the innate immune system's response to infection by leveraging previous exposure to microbial products. This approach is promising for protecting vulnerable patients. A study by Owen et al. investigated the mechanism by which the TLR4 agonist monophosphoryl lipid A generates trained immunity in macrophages. The authors found that TLR4-mediated trained immunity relies on myeloid differentiation primary response 88 (MyD88), but not on Toll/interleukin-1 receptor domain-containing adaptor protein inducing interferon-beta (TRIF). The TLR4-activated immune response provided macrophage-dependent protection against *Staphylococcus aureus* infection for at least 2 weeks. Activation of endosomal TLRs could generate sufficient antiviral immune responses. Indeed, antiviral efficacy upon activation of TLR7 and/or TLR8 agonists has been studied by many groups, including ours. A modification of this strategy was investigated by Vlaming et al., by comparing the antiviral efficacy of various combinations of different TLR7 and/or TLR8 agonists with retinoic acid-inducible gene I-like receptor (RLR) agonists. They found that co-stimulating the TLR8 and RLR pathways produced a stronger antiviral immune response compared to activating TLR8 alone, due to increased production of IL-12p70 and type I interferons. Next, house dust mites frequently cause allergic airway diseases in tropical urban environments, where the protein parts of the dust mites (i.e., Der p1 and Der p2) are known to generate the production of specific IgE and induce cross-linking of the IgE-bound to mast cells or basophils, resulting in an allergic immune response. Busold et al. compared the protein and non-protein components of house dust mites in activating dendritic cells to trigger adaptive allergic immune responses. Notably, the non-protein parts were responsible for the maturation of dendritic cells and the subsequent release of cytokines such as IL-6 and IL-12. This activation was also found to be dependent on TLR4 and spleen tyrosine kinase (Syk). Additional insight into the TLR signaling pathway during immune responses was gained in a review by Pereira and Gazzinelli, in which they described the role of IL-1R-associated kinase (IRAK) proteins in the formation, stabilization, and disassembly of the myddosome, a supramolecular organizing center that includes MyD88, IRAK, and TNF receptor-associated factor (TRAF) proteins. The second group of articles includes publications on modulating TLR activation to induce immune tolerance. TLR2, TLR4 and TLR5 are the main extracellular receptors on gut epithelial cells that identify potential PAMPs in the intestinal lumen. It is important to understand how TLR signaling promotes immune tolerance, as establishing an immune-tolerant environment in the gastrointestinal tract is vital for normal physiological function. In an interesting study by Nanthakumar et al., fucosylated TLR4 (but not the non-fucosylated TLR4) was important to convert sialylated glycans to fucosylated glycans on the surface of intestinal epithelial cells. The fucosylated intestinal mucosa supports a microbial community of fucotrophic microbes that enhance the host resistance to various mucosal challenges. Indeed, the activation of fucosylated TLR4 triggered a non-inflammatory signaling cascade (dependent on ERK and JNK but independent of NF-κB), leading to increased expression of the *fucosyltransferase 2* gene in intestinal epithelial cells. Various probiotics have also been investigated for their ability to improve immune tolerance in the gastrointestinal tract. Haque et al. studied the role of probiotic *Lactobacillus acidophilus* in improving the intestinal epithelial tight junction barrier, which is important to prevent inflammatory bowel disease. The specific strain of *Lactobacillus acidophilus* (i.e., LA1) significantly reduced the increase in intestinal tight junction permeability upon exposure to TNF-alpha. The LA1 strain achieved this by suppressing NF-kB activation through a TLR2-dependent but MyD88-independent mechanism in intestinal epithelial cells. Finally, Jia et al. investigated various immunomodulatory combinations (including TLR agonists) for the generation of tolerogenic dendritic cells, as these cells could be used as a potential therapy for autoimmune diseases and transplantation rejection. The authors demonstrated that two combinations successfully generated tolerogenic dendritic cells, as indicated by elevated interleukin-10 and reduced TNF-alpha secretion in murine bone marrow-derived dendritic cells. The combinations were AD80 (a multi-kinase inhibitor) paired with Pam2 (a TLR2 agonist) and with CEP-33779 (a JAK inhibitor) combined with R848 (a TLR7 and TLR8 agonist), suggesting a strategy to develop tolerogenic dendritic cells *ex vivo*.

In summary, these original articles and a review offered new insights into TLR signaling in the activation of immune responses and induction of immune tolerance. These publications may encourage further exploration of the molecular mechanisms involved in TLR signaling and the health effects of co-stimulating TLRs with other pattern-recognition receptors. Such studies are needed to translate scientific discoveries into clinical practice for the treatment of patients with immune disorders.

